# Neuralgia Facial, or Tic Douloureux

**Published:** 1856-01

**Authors:** T. D. Thompson

**Affiliations:** Providence, Rhode Island


### NEURALGIA FACIAL, OR TIC DOULOUREUX.

BY T. D. THOMPSON, D. D. S., PROVIDENCE, R. I.

It is not surprising that many individuals should be found suffering from this painful disease. The reasons are obvious to every properly instructed dentist. Considering the nervous connections of the teeth, their wide-spread sympathetic relations, and realizing the fact likewise that many of the organs remote from them nervously sympathize, how can it be expected otherwise than that this disease should be thus common, when the teeth are so often found in an impaired and diseased condition?

Considering the frequency of this disease, it has to us been a matter of surprise that so little information existed in regard to it. This want of information is not confined to the non-professional. The treatment of this disease by many of our physicians appears to be in most instances for pure neuralgia, overlooking or not suspecting the true cause, which, in the large majority of cases, is attributable to the diseased state of the teeth. Many physicians seem to think the teeth of minor importance, regarding them apparently as parts of the system, insulate. Such individuals have studied, we think, the anatomical and physiological principles of the human system to little, or no purpose.

The preceding reflections seem to be pertinent to the case we wish to introduce to your readers.

A lady residing in the city of Providence, between the ages of forty and fifty, of full habit, was recently brought under a severe neuralgic affection of the face and sympathizing parts. The premonitory symptoms were occurring at intervals for some time previous to the general attacks. The disease in the case of this individual was at length brought to a severe crisis; she was prostrated, unable to do anything. She was attended three or four weeks by her physician, during which time she became very much reduced in physical strength—so much so, as to create fears in regard to her life. Her relatives in a distant place were telegraphed of her situation, and the necessity of their immediate presence. At this point in her case, her physician stated he had done all in his power for her relief, and withdrew. Another physician was then called. This was the situation of the case when I obtained information of it. Being acquainted with the lady, having sometime previous extracted some teeth for her, I recollected that there were several roots of teeth remaining in the superior maxillary, and at once suspected that her suffering must arise in part, if not wholly, from them. I immediately called upon the family and stated my opinion in regard to the case; they readily admitted that it might be as I suspected. I saw the lady, and expressed to her my views; she likewise was somewhat of my opinion. Notwithstanding she being very low, I proposed to extract the troublesome roots; she expressed her willingness, provided she could inhale ether. I told her if her then attending physcian thought advisable, I would comply with her wishes. On the day following, I was called upon by her daughter and requested to wait upon her mother and extract the roots, her physician consenting that ether should be administered.

I accordingly waited upon the lady, administered the ether, and extracted four roots, namely, one left superior cuspid and bicuspid, one right superior lateral incisor, one left superior second molar. The result of the operation, in connection with medical treatment, in two weeks was such, that the lady was up and out of her room, and gaining bodily strength.

One feature in this case remains to be noticed, and one that should be impressed upon the mind of every reader, namely, the entire loss of sight to the individual. She was unable to distinguish any object whatever. Had the exciting cause in this case been understood at first, and the roots of teeth been promptly removed, the great suffering that this individual was subjected to would have been avoided. Her loss of sight, which to her now is a source of great mental suffering, would not in all probability have occurred. The latest information I have received respecting this interesting case was, that the lady was in Boston, under treatment for her sight. Her physician, I understood, had but little hopes that she would recover it.—*American Journal.*

